# A guide to Whittle maximum likelihood estimator in MATLAB

**DOI:** 10.3389/fnetp.2023.1204757

**Published:** 2023-10-31

**Authors:** Clément Roume

**Affiliations:** IRIMAS UR UHA 7499, University of Haute-Alsace, Mulhouse, France

**Keywords:** fractals, Whittle’s likelihood, tutorial, fractional Gaussian noise, ARFIMA (0,d,0)

## Abstract

The assessment of physiological complexity via the estimation of monofractal exponents or multifractal spectra of biological signals is a recent field of research that allows detection of relevant and original information for health, learning, or autonomy preservation. This tutorial aims at introducing Whittle’s maximum likelihood estimator (MLE) that estimates the monofractal exponent of time series. After introducing Whittle’s maximum likelihood estimator and presenting each of the steps leading to the construction of the algorithm, this tutorial discusses the performance of this estimator by comparing it to the widely used detrended fluctuation analysis (DFA). The objective of this tutorial is to propose to the reader an alternative monofractal estimation method, which has the advantage of being simple to implement, and whose high accuracy allows the analysis of shorter time series than those classically used with other monofractal analysis methods.

## 1 Introduction

For nearly three decades, numerous studies have shown that invariant-scale structures appear in biological signals. These studies have widely suggested that this type of structure, also called fractal structures, hints at the complexity of the system that produced the signal (Peng et al., 1994; Hausdorff et al., 1996; Ivanov et al., 1998; Goldberger et al., 2002; Kello et al., 2007; Delignieres and Torre, 2009; Harrison and Stergiou, 2015; Vaz et al., 2020). Formally, scale invariance can be described in several ways. In the time domain, a signal is scale-invariant when its statistical properties are consistent across scales $y\left(ct\right)≜c^{H}y\left(t\right)$ , with ≜ denoting equality of statistical distribution, whereas in the spectral domain, stationary fractal signals decay as $S\left(f\right)\propto 1/f^{2H-1}$ . In both cases, the signals are characterized by the Hurst exponent *H*, which defines the quality of the fractal structure. Thus, signals whose *H* is between 0.5 and 1 are considered persistent or long-term memory processes. Signals whose *H* is between 0 and 0.5 are anti-persistent or short-term processes. Finally, signals whose *H* is equal to 0.5 are random and can be assimilated to white Gaussian noise. The objective of fractal analysis is to estimate the value of *H*.

The estimation of the *H* exponent has become very relevant in the fields of health and aging because this fractal structure tends to diminish or even disappear with the first signs of age or disease, making the exponent *H* a very promising predictor. We (Almurad et al., 2017; Almurad et al., 2018; Ezzina et al., 2021) have also suggested, through several studies, that it was possible to restore complexity in older adults. However, all the participants in our studies were characterized by healthy aging and did not have any motor disability limiting their movements. Fractal analyses require long signals of more than 500 data points to ensure that the estimated *H* value is reliable, which, in the case of walking, represents an analysis time of 10–12 min (Phinyomark et al., 2020). It is obvious that for people whose age has a greater impact on their motor skills or who have diseases limiting their motricity, it is impossible to walk for such a long time. One can believe that it is necessary to develop a more precise method of analysis that would allow reducing the duration of the experimental process.

Among the mathematical models of long-memory processes, two models have been widely used to construct methods for estimating the *H* exponent. The first method, proposed by Mandelbrot and Van Ness (1968), is the fractional Gaussian noise (fGn) and fractional Brownian motion (fBm) model. It is the first fractal process model that has been formulated, describing both stationary (fGn) and non-stationary (fBm) processes. In this case, stationarity corresponds to a process with constant mean and variance, and non-stationarity corresponds to a process with a variance dependent on time and *H*. The second model, formulated by Granger and Joyeux (1980) and Hosking (1981), is the ARFIMA (p,d,q) model. This model allows the description of short memory processes (via MA and AR component) AND long memory processes (via FI component). In the context of this paper, we focus only on the long-memory FI component. The model used here is therefore an ARFIMA (0,*d*,0). Unlike the fBm/fGn model, the ARFIMA (0,*d*,0) model holds only for stationary processes. However, in the spectral domain, ARFIMA (0,*d*,0) and fGn have an equivalent spectral decay, which makes it possible to compare exponents *d* and *H* via the d=H−1/2 conversion.

One can note that the Hurst exponent H does not allow distinction between stationary fGn and non-stationary fBm processes. Concretely, white Gaussian noise and an ordinary Brownian motion share the same exponent H equal to 0.5. On the other hand, if *a priori*, the ARFIMA (0,*d*,0) model applies only to stationary processes, it is still possible to estimate *d* from the increments of non-stationary processes (Diebolt and Guiraud, 2005), so *d* suffers from the same distinction issue. Yet, in the spectral domain, stationary and non-stationary processes seem to follow a continuum, as illustrated by the continuity of the spectral exponent (Halley and Inchausti, 2004). Detrended fluctuation analysis (DFA) (Peng et al., 1995), for example, is built around this assumption, and the exponent α introduced into this analysis ranges from 0 to 1 for stationary processes and from 1 to 2 for non-stationary processes. In concrete terms, α=H for fGn, α=H+1 for fBm, and α=d+1/2 for ARFIMA (0,*d*,0). Thus, considering the previous example, in the α metric, white Gaussian noise is characterized by an exponent α=0.5 , while an ordinary Brownian motion is characterized by an exponent α=1.5 . Since we aim to build an algorithm that operates without any *a priori* assumptions about the stationarity of time series, the output estimate will be expressed in the α metric.

In addition to the series size issues mentioned previously, Beran et al. (2013) add that heuristic/graphical methods, such as DFA (Peng et al., 1995), “*are easy to implement and may serve as descriptive tools and a first heuristic check, there are many reasons for using more sophisticated methods when it comes to actual statistical inference.”* Among these more sophisticated methods are maximum likelihood estimators (MLEs) and, particularly, Whittle’s method. Whittle’s MLE is a parametric estimator based on an optimization problem. Beran suggests that this estimator, like the classical MLE, is consistent. The choice of Whittle’s MLE over the classical MLE has two explanations. The first is computational: the complexity of the classical MLE is $O\left(N^{2}\right)$ , while that of Whittle approximation is $O\left(N\log_{2}N\right)$ . The second is practical: for values of *H* close to 1, the covariance matrix to be inverted in MLE is close to the singularity, which can generate computational errors.

In a previous study (Roume et al., 2019), we had already suggested that Whittle’s MLE allows a better estimation of the *H* exponent than DFA and the spectral analysis. However, the algorithm we used, the ARFIMA (p,d,q) estimator proposed by G. Intzelt on the MathWorks File Exchange platform^1^, went far beyond the analysis of long-memory processes, allowing, for example, the addition of short-term components, forecasting, and signal generation. We therefore propose the present tutorial, which allows a simpler implementation of Whittle’s approximation of MLE, focusing only on the long-term dependencies.

The purpose of this tutorial is to provide a step-by-step guide to construct this analysis method similar to the method used by Ihlen in his tutorial for MF-DFA (Ihlen, 2012). In addition, at each step, we describe the formal aspects underlying the construction of the algorithm. We also propose to the reader a single-file and standalone algorithm to facilitate its use and diffusion. Then, to facilitate reading, we have used a different font for the variables, parameters, and commands used in MATLAB. We suggest the reader to download the code files and datasets deposited in a GitHub repository at https://github.com/clementroume/Whittle_maximum_likelihood_estimator_tutorial and add the downloaded folder to the MATLAB path to follow this tutorial. The remainder of this article is organized as follows: Section 2, *Whittle’s maximum likelihood estimator in MATLAB*, gives the step-by-step construction method of the whittle.m algorithm. Meanwhile, Section 3, *Whittle’s maximum likelihood performances*, outlines a complete benchmark of this algorithm against DFA.

## 2 Whittle’s maximum likelihood estimator in MATLAB

This method of analysis is an optimization problem, and the principle is to estimate the value of the Hurst exponent $\hat{H}$ which maximizes Whittle’s log-likelihood function $l_{W}\left(H\right)$ :

$$l_{W}\left(H\right)=-\frac{2}{N}\sum_{j=1}^{m}\left(\ln cT^{\prime }\left(\omega_{j};H\right)+\frac{P\left(\omega_{j}\right)}{cT^{\prime }\left(\omega_{j};H\right)}\right),$$ (1)

where

• m is the integer part of $\left(N-1\right)/2$ .
• $\omega_{j}$ are the Fourier frequencies defined as ω=2πj/N .
• $P\left(\omega_{j}\right)$ is the periodogram of the observation vector $x\left(j\right)$ of length N .
• $T’\left(\omega_{j};\;H\right)$ is the theoretical power spectral density of the model process, with parameter H .
• c is a constant of proportionality used to adjust the power $T’\left(\omega_{j};\;H\right)$ to that of $P\left(\omega_{j}\right)$ .
• H is the Hurst exponent belonging to $]0,1[$ .

Whittle’s log-likelihood function is an approximation of the likelihood function for stationary Gaussian processes, in this case, fGn or ARFIMA (0,*d*,0). As further illustrated in Figure 5, the principle is to construct the $l_{W}\left(H\right)$ function over the interval ]0,1[ from the periodogram *P* of the signal and the theoretical power spectral density *T′* of the chosen theoretical model. The main characteristic of the $l_{W}\left(H\right)$ function is that it reaches its maximum for the $\hat{H}$ value characterizing the analyzed signal.

We will detail this method through seven sections: Section 2.1, *Periodogram power spectral density estimate*, introduces the computation to estimate the periodogram of the signal. Section 2.2, *Theoretical power spectral density of the model process*, is a sub-step presenting the two possible alternatives in the choice of the theoretical spectrum. Section 2.3, *Fitting the power of the model process to that of the signal*, is a sub-step where the constant c is computed. Section 2.4, *Whittle’s log-likelihood function*, describes the computation of Equation 1. Section 2.5, *Resolving the optimization problem*, introduces the method to find the maximum of Whittle’s log-likelihood function. Section 2.6, *The case of non-stationary signals*, proposes a method to detect non-stationary signals (i.e., with H > 1 ) and estimate their fractal exponent. Finally, Section 2.7, *The construction of whittle.m algorithm*, presents the order in which the various code sections must be arranged to obtain the whittle.m all-in-one algorithm. Each step will be represented first in the mathematical formalization and then as a MATLAB code. Finally, we would like to clarify how the different exponents *H*, *d*, and α are used in the seven sections of this tutorial. The first four sections consist of the construction of Whittle’s function, on the one hand, with the fBm/fGn model, and, on the other hand, with the ARFIMA (0,d,0) model, so these parts are presented around the two exponents *H* and *d*. From Section 2.5 onwards, we introduce the use of the α exponent, which allows the algorithm to analyze stationary and non-stationary processes without prior distinction. However, to maintain readability between the two models, we have named the two output variables Afgn for the value of α estimated from the fGn/fBm model and Aarf for the value of α estimated from the ARFIMA (0,d,0) model.

Before beginning this guide, the reader can type load fractalsignals. mat in the MATLAB command window to load the time series: choleskyfgn, arfima0d0, whitenoise, and empirical. These signals will be used all along this guide to illustrate each step of the construction of the algorithm. choleskyfgn is a simulated exact fGn signal with an H exponent equal to 0.8, and it was generated via the Cholesky decomposition method. arfima0d0 is a simulated ARFIMA (0,*d*,0) process with a d exponent equal to 0.3, which is equivalent to a fGn with H=0.8 . whitenoise is a normally distributed random noise signal, which was generated with the following MATLAB command: whitenoise = normrnd (0.1, [1024.1]). empirical is a signal retrieved from the study by Almurad et al. (2018), and it corresponds to step-to-step timing in an arm-in-arm synchronized walking task. Please consider that all the lines of code presented in this tutorial have been written and tested under MATLAB version 2021b. Although most of the codes work regardless of the version of the software application, some recent functions like nexttile, introduced with MATLAB 2019b, could cause compatibility problems and should be replaced by the subplot function.

### 2.1 Periodogram power spectral density estimate

The first step is to estimate the power spectral density of the observation vector. This estimation can be carried out by calculating the periodogram of the signal corresponding to the squared modulus of the discrete Fourier transform of the signal. The periodogram is formally written as

$$P\left(\omega_{j}\right)=2*\left(\frac{1}{2\pi N}{\left|\sum_{j=1}^{m}x\left(j\right)e^{-i\omega_{j}j}\right|}^{2}\right),\frac{2\pi }{N}<\omega_{j}<\frac{2\pi m}{N},$$ (2)

where the set of variables used in Equation 2 is the same as that described in Equation 1. To meet the requirements of Equation 1, we note that the frequency range ω is limited. The frequency ω=0 is excluded, and the maximum frequency is ω=2πm/N , where m is the integer part of $\left(N-1\right)/2$ . When the length of the signal is equal to the power of 2, this procedure excludes the Nyquist frequency; otherwise, it limits the length of the spectrum to half the length of the signal minus 1. Finally, note that the value of the periodogram is doubled. This is a method described at the end of the help paragraph of the periodogram() function on the MathWorks website^2^, which conserves the total power in one-sided periodograms. The following MATLAB codes are used to estimate the periodogram of the signal:

…………………………………………………………
**MATLAB code 1**: Periodogram estimation.
X=zscore(x); N=length(X);
m=floor((N-1)/2);
[Pxx,wxx]= periodogram(X); P=Pxx((2:m+1));
w=wxx((2:m+1));
…………………………………………………………

The first line standardizes the observation vector x by setting its mean to 0 and its standard deviation to 1. This operation is relatively common in the field and is essential for the rest of this tutorial because Equations 3, 4 in the following sections are derived from the autocorrelation function (and not from the autocovariance function) and therefore assume a variance equal to 1. This operation also normalizes the total power of the spectrum *P*, but it does not change the shape of the spectrum, so it does not alter the information of the fractal exponents.

**FIGURE 1:**
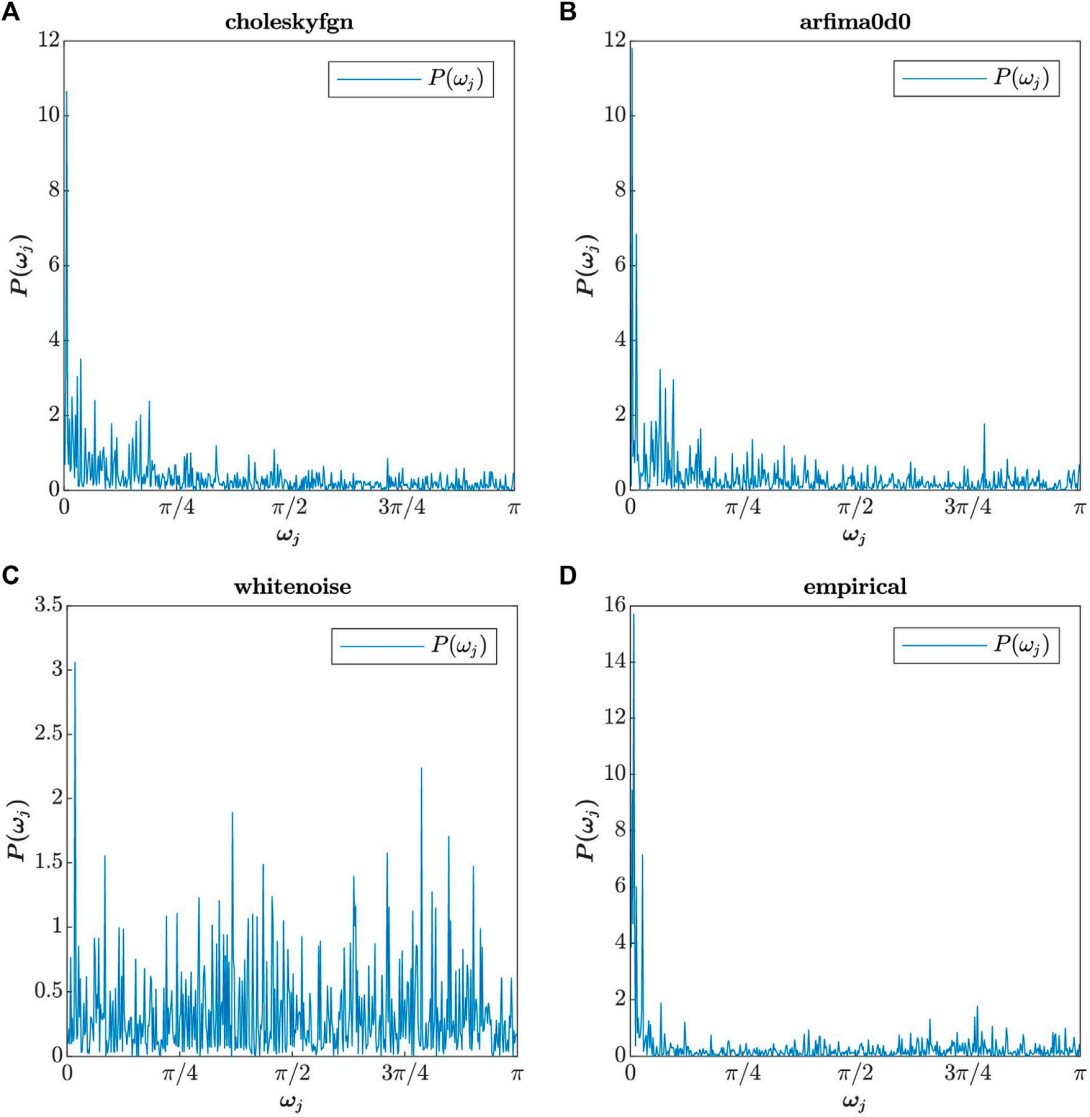
Estimation of power spectral density via the periodogram method for choleskyfgn **(A)**, arfima0d0 **(B)**, whitenoise **(C)**, and empirical **(D)** signals.

The second line returns the length N of the input signal x, and the third computes the upper bound m. In the fourth line, we use the Signal Processing Toolbox command periodogram() to estimate the power spectral density of the input signal x, and an alternative code using the fft() command is given if the periodogram() command is not included in your MATLAB version. Finally, the fifth and the sixth lines bound P and w within the interval presented in Equation 2. Figure 1 represents the plot of the estimated periodograms of the series choleskyfgn, arfima0d0, whitenoise, and empirical.

Type Fig1_PSD in the command window to access Figure 1.

…………………………………………………………
**MATLAB code 1bis**: Periodogram estimation using fast Fourier transform
X = zscore(x); N = length(X);
m = floor ((N-1)/2);
Y = fft(X);
P=(1/(pi*N))*abs (Y (2:m+1)′). ^2;
w=(2*pi*(1:m)′)/N
…………………………………………………………

### 2.2 Theoretical power spectral density of the model process

In this sub-step, we present the equations and their computation for the two theoretical spectral densities derived from the fGn/fBm and ARFIMA (0,*d*,0) models, respectively.

The theoretical fGn spectral density was given by Li and Lim (2006) as follows:

$$T_{fGn}^{\prime }\left(\omega_{j};H\right)=\sin\left(H\pi \right)\Gamma \left(2H+1\right){\left|\omega_{j}\right|}^{1-2H},$$ (3)

where Γ is the gamma function, and the other variables used in Equation 3 are the same as those described in Equation 1. The following MATLAB code is the computation of Equation 3.

…………………………………………………………
**MATLAB code 2**: Theoretical power spectral density of the fGn process
Tp = sin (pi*H)*gamma ((2*H)+1)*(abs(w).^(1-(2*H)));
…………………………………………………………

The theoretical ARFIMA (0,*d*,0) spectral density was given by Taqqu et al. (1995) as follows:

$$T_{ARFIMA}^{\prime }\left(\omega_{j};d\right)=\frac{1}{2\pi }{\left(2\sin\frac{\omega_{j}}{2}\right)}^{-2d},$$ (4)

where *d* is the integration parameter belonging to $]-0.5,0.5[$ . One can easily convert the exponent H to the exponent d via the equation d=H−0.5 . The following MATLAB code converts H to d and computes Equation 4.

…………………………………………………………
**MATLAB code 3**: Theoretical power spectral density of the ARFIMA (0,d,0) process
d = H-0.5;
Tp=(1/(2*pi))*(2*sin (w/2)).^-(2*d);
…………………………………………………………

**FIGURE 2:**
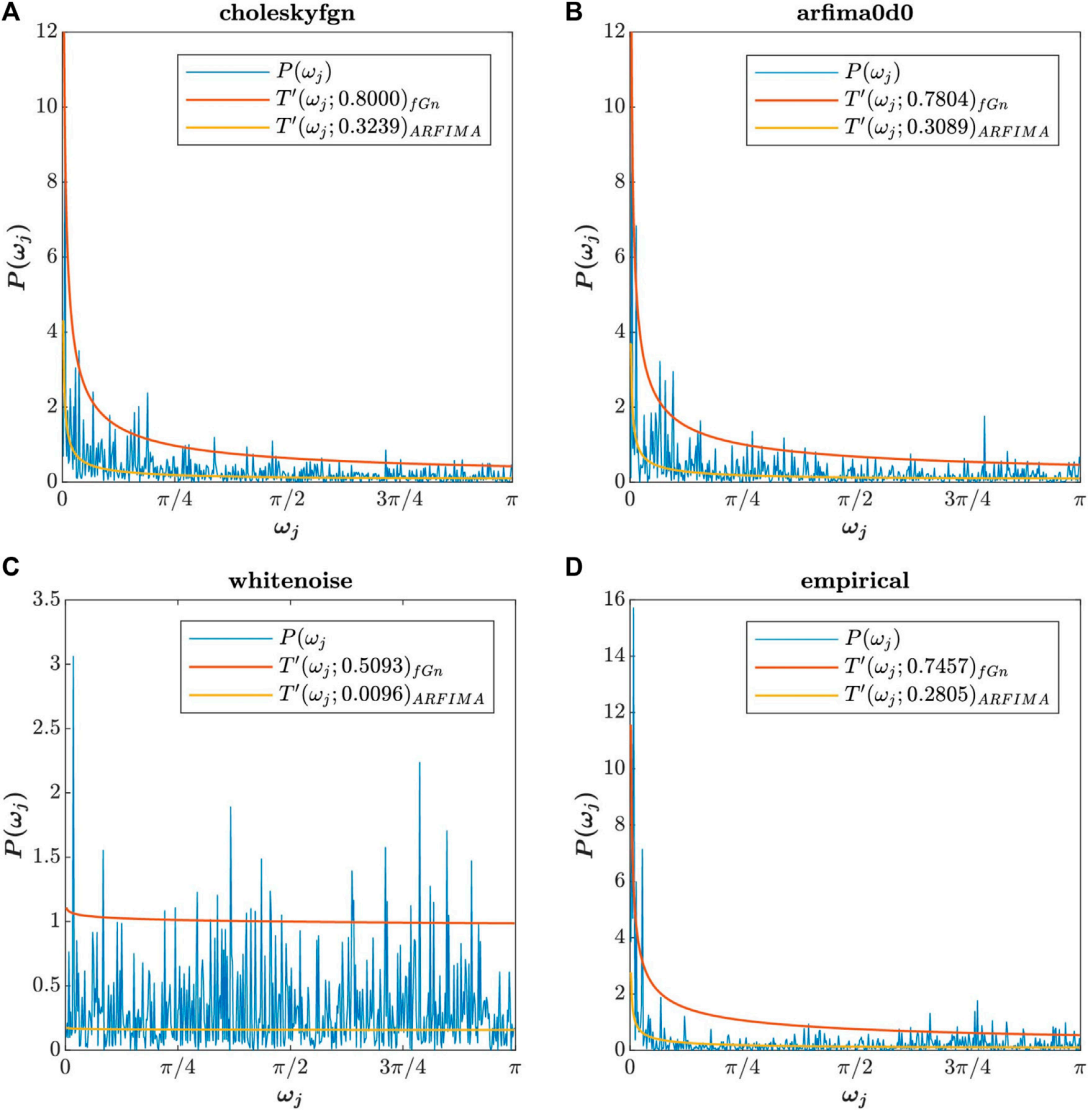
Periodogram of choleskyfgn **(A)**, arfima0d0 **(B)**, whitenoise **(C)**, and empirical **(D)** signals with the theoretical power spectral density of fGn (orange curve) and ARFIMA (0,*d*,0) (yellow curve). The theoretical power spectral densities were computed with the estimated values of *H* and *d* obtained via whittle.m. Those values, entered in MATLAB code 2 and 3, are presented in Table 1.

Figure 2 repeats Figure 1 by adding the theoretical spectral densities calculated using MATLAB codes 2 and 3. To better illustrate the global functioning of the algorithm, in Figure 2, we present the theoretical power spectral densities computed with the estimated values of *H* and *d* obtained via whittle.m. These values are reported in Table 1.

**TABLE 1:** *H* and *d* values of choleskyfgn, arfima0d0, whitenoise, and empirical signals estimated via whittle.m.

	Signal			
	choleskyfgn	arfima0d0	whitenoise	empirical
*H*	0.8000	0.7804	0.5093	0.7457
*d*	0.3239	0.3089	0.0096	0.2805

Type Fig2_TPSD in the command window to access Figure 2.

### 2.3 Fitting the power of the model process to that of the signal

As advised by Jennane et al. (2001), in this sub-step, we calculate the constant of proportionality c to adjust the power of the model process to that of the signal as follows:

$$c=\frac{\sum_{\omega }P\left(\omega_{j}\right)}{\sum_{\omega }T^{\prime }\left(\omega_{j},H\right)}.$$ (5)

…………………………………………………………
**MATLAB code 4**: Fitting theoretical spectrum
c = sum(P)/sum (Tp);
T = c*Tp;
…………………………………………………………

**FIGURE 3:**
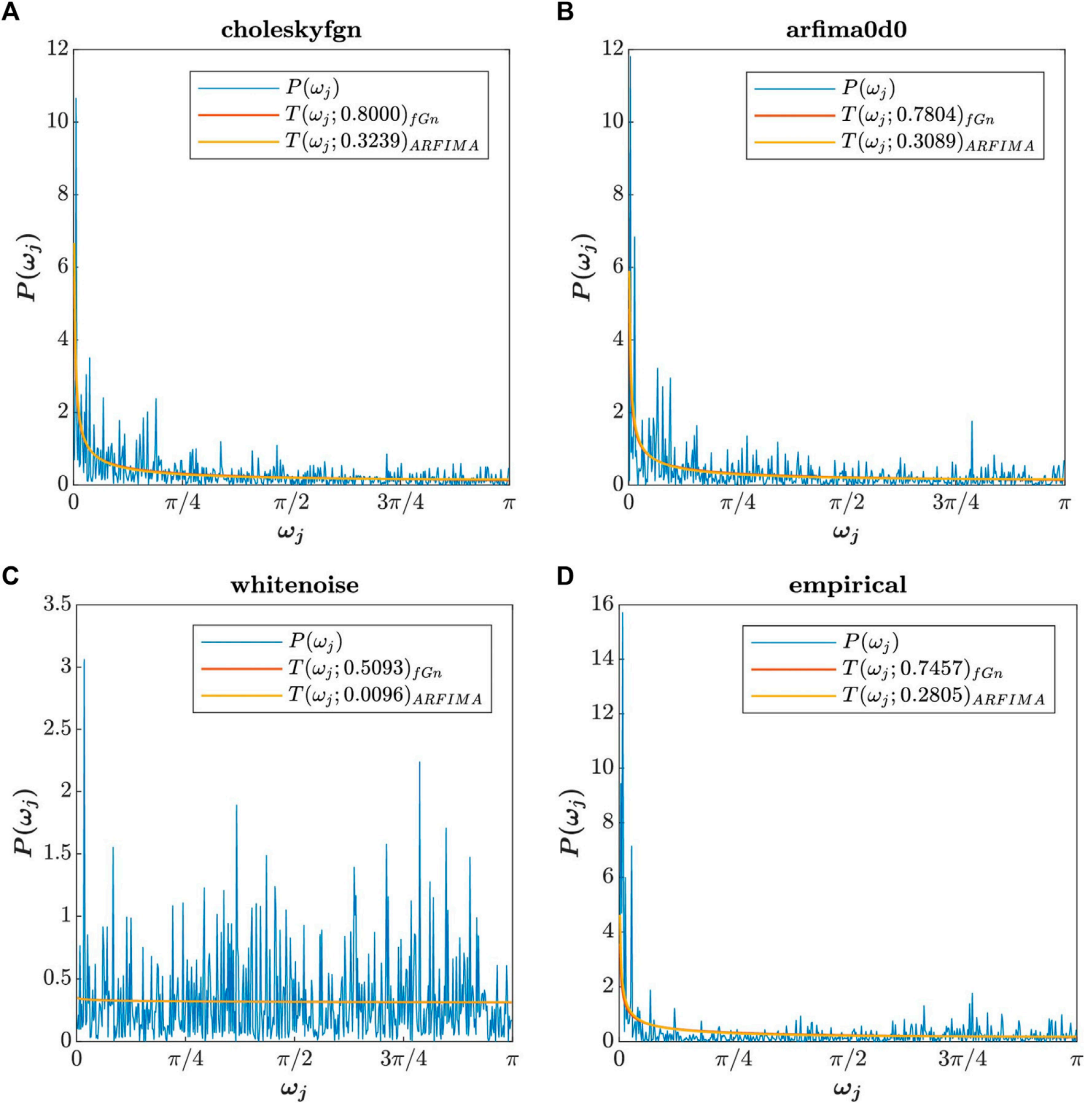
Periodogram of choleskyfgn **(A)**, arfima0d0 **(B)**, whitenoise **(C)**, and empirical **(D)** signals (blue curve) with the adjusted theoretical power spectral density of fGn (orange curve) and ARFIMA (0,*d*,0) (yellow curve). The *H* and *d* values are the same as those used in the previous figure.

In the first line, the constant c is calculated, and in the second line, the theoretical spectrum Tp is adjusted to the empirical periodogram P. Figure 3 shows the plot of Figure 2 with adjusted theoretical spectrum. The total power of the theoretical spectrum Tp is determined by the value of *H* in Equation 3 or *d* in Equation 4, but the function of fractal exponents is to shape the spectrum, not to modulate its power, hence the need for adjustment between theoretical power and signal power.

Type Fig3_TPSD_adjusted in the command window to access Figure 3.

### 2.4 Whittle’s log-likelihood function

In this second step, we construct Whittle’s log-likelihood function by injecting the two previous sub-steps into Equation 1, which is the function that we want to maximize. However, the optimization toolbox of MATLAB only allows minimizing functions, so we will have to minimize the inverse of Equation 1. This inverse function is written in the MATLAB code as follows:

…………………………………………………………
**MATLAB code 5**: Whittle’s log-likelihood function
lwH=(2/N)*sum (log(T)+(P./T));
…………………………………………………………

where lwH is the inverse of Whittle’s likelihood function, N is the length of the observation vector, T is the scaled theoretical periodogram computed via MATLAB codes 2 or 3 and then MATLAB code 4, and P is the estimated periodogram of the observation vector computed via MATLAB code 1. MATLAB codes 6 and 7 are given in the following sections, which are the functions declared in MATLAB and which we will have to minimize. These codes correspond to the combination of MATLAB codes 2–5. MATLAB code 6 gives the function based on the theoretical spectrum of fGn, while MATLAB code 7 gives the function based on the theoretical spectrum of ARFIMA (0,*d*,0). These functions will have to be placed either at the end of the mother code whittle.m (as is the case with the provided code) or in two separate files that can be named WLLFfgn.m and WLLFarf.m.

…………………………………………………………
**MATLAB code 6**: Whittle’s log-likelihood function with fGn theoretical PSD
function lwHfgn = WLLFfgn (H,w,P,N)
Tp = sin (pi*H)*gamma ((2*H)+1)*(abs(w).^(1-(2*H)));
c = sum(P)/sum (Tp);
T = c*Tp;
lwHfgn=(2/N)*sum (log(T)+(P./T));
…………………………………………………………
**MATLAB code 7**: Whittle’s log-likelihood function with ARFIMA (0,d,0) theoretical PSD
function lwHarf = WLLFarf (H,w,P,N)
d = H-0.5;
Tp=(1/(2*pi))*(2*sin (w/2)).^-(2*d);
c = sum(P)/sum (Tp);
T = c*Tp;
lwHarf=(2/N)*sum (log(T)+(P./T));
…………………………………………………………

In the first line, the function is used to declare the functions WLLFfgn and WLLFarf. The outputs are lwHfgn for code 6 and lwHarf for code 7 and correspond to $l_{W}\left(H\right)$ in Equation 1. The inputs are as follows: H is the Hurst exponent, w is the Fourier frequencies, P is the estimated periodogram of the observation vector, and N is the length of the observation vector.

**FIGURE 4:**
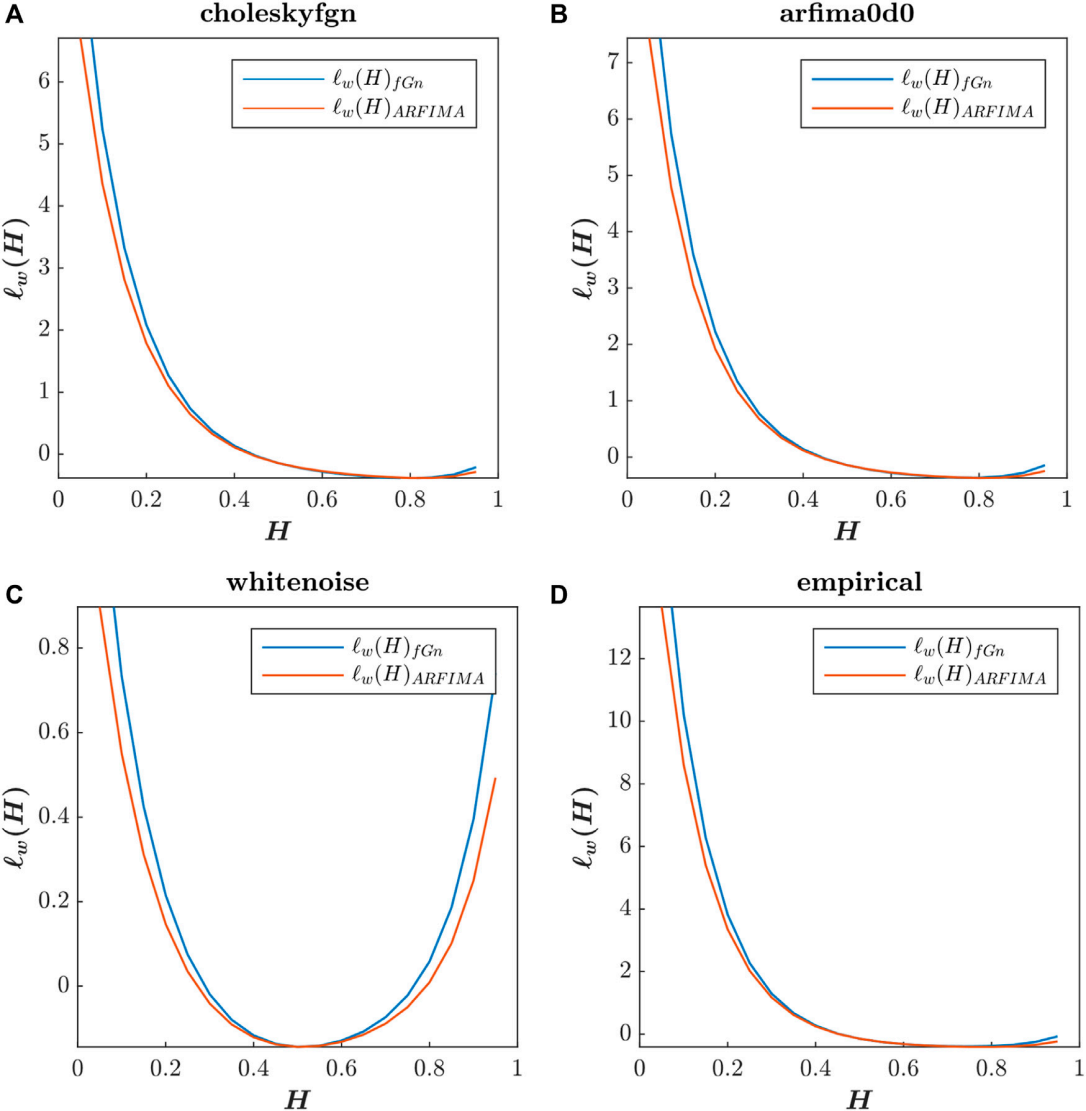
Whittle’s log-likelihood functions of choleskyfgn **(A)**, arfima0d0 **(B)**, whitenoise **(C)**, and empirical **(D)** signals. The blue curves correspond to Whittle’s likelihood function calculated using the fGn theoretical spectrum, while the orange curves correspond to the same function calculated using the ARFIMA (0,*d*,0) theoretical spectrum.

In Figure 4, we present the plots corresponding to these two functions for our four test signals: choleskyfgn, arfima0d0, whitenoise, and empirical, with H values ranging from 0.05 to 0.95 by steps of 0.05.

Type Fig4_lwH in the command window to access Figure 4.

### 2.5 Resolving the optimization problem

In this third step, we describe the method to solve the optimization problem. In other words, we must find the value of H for which we reach the minimum of the inverse of Whittle’s likelihood:

$$\hat{\alpha }=\underset{0<H<1}{\mathrm{argmin}}\left\{\frac{2}{N}\sum_{j=1}^{m}\left(\ln cT^{\prime }\left(\omega_{j};H\right)+\frac{P\left(\omega_{j}\right)}{cT^{\prime }\left(\omega_{j};H\right)}\right)\right\},$$ (6)

where $\hat{\alpha }$ is the estimate of the fractal exponent of the observation vector $x\left(j\right)$ . As stated in the introduction of this first part, the present section 2.5 marks the transition from the exponents *H* and *d* characterizing the stationary processes fGn and ARFIMA (0,*d*,0) to the exponent α that can characterize the full set of stationary and non-stationary processes on a continuum from α=0 to α=2 . The α metric allows the whittle.m algorithm to work without making the *a priori* distinction between stationary and non-stationary signals, as DFA does.

In order to implement the operation of Equation 6, we use the MATLAB function fminbnd(), which is a minimizer based on golden section search and parabolic interpolation. The MATLAB codes to find the minimum of the WLfgn and Wlarf functions are as follows:

…………………………………………………………
**MATLAB code 8**: Optimization for fGn-based Whittle’s log-likelihood function
Afgn = fminbnd (@(H) WLLFfgn (H,w,P,N),0,1);
…………………………………………………………
…………………………………………………………
**MATLAB code 9**: Optimization for ARFIMA (0,d,0)-based Whittle’s log-likelihood function
Aarf = fminbnd (@(H) WLLFarf (H,w,P,N),0,1);
…………………………………………………………

Afgn and Aarf are the two values of $\hat{\alpha }$ ; the first one is estimated by fGn-based Whittle’s likelihood, and the second is estimated by ARFIMA-based Whittle’s likelihood. The syntax @(H) intimates the function fminbnd() that *H* is the variable to be optimized, while WLfgn (H,w,P,N) and WLarf(H,w,P,N) are the functions that are optimized (corresponding to MATLAB codes 6 and 7). Finally, 0 is the lower bound and 1 is the upper bound in the optimization problem. This algorithm never optimizes on the bounds, so using 0 as a lower bound and 1 as an upper bound satisfies both theoretical conditions 0<H<1 and −0.5<d<0.5 .

### 2.6 The case of non-stationary signals

By definition, Whittle’s log-likelihood function only holds for stationary signals such as fGn or ARFIMA (0,*d*,0). As a result, when analyzing non-stationary signals, the minimum of this function occurs when $\hat{\alpha }$ is almost equal to 1. More precisely, when we refer to the help provided with the fminbnd() function^3^, this translates into values of $\hat{\alpha }$ greater than $\left(1-2.10^{-4}-6\sqrt{2,2204\times 10^{-16})}∼0.9998\right.$ . Thus, if the algorithm returns a value greater than 0.9998, we can classify the signal as non-stationary. To calculate the fractal exponent for the non-stationary signals, we apply the method proposed by Diebolt and Guiraud (2005), which consists in analyzing a differentiated version of the signal, and then add 1 to $\hat{\alpha }$ . This translates in MATLAB code as

…………………………………………………………
**MATLAB code 10**: If the observation vector is non-stationary, fGn-based Whittle’s likelihood
if Afgn >= 0.9998
[Pyy,wyy]= periodogram (diff(X));
mdiff = floor ((N-2)/2);
Pdiff = Pyy ((2:mdiff+1));
wdiff = wyy ((2:mdiff+1));
Afgn = fminbnd (@(H) WLLFfgn (H,wdiff, Pdiff, (N-1)),0,1)+1;
end
…………………………………………………………
**MATLAB code 11**: If the observation vector is non-stationary, ARFIMA-based Whittle’s likelihood
if Aarf >= 0.9998
[Pyy,wyy]= periodogram (diff(X));
mdiff = floor ((N-2)/2);
Pdiff = Pyy ((2:mdiff+1));
wdiff = wyy ((2:mdiff+1));
Aarf = fminbnd (@(H) WLLFarf (H,wdiff, Pdiff, (N-1)),0,1)+1;
end
…………………………………………………………

The first line of both codes is the logical test. If the answer is true, then the four consecutive lines are computed. In the second line, the periodogram is estimated on the differentiated observation vector diff(x). In the third line, mdiff is calculated because diff(x) is one point shorter than x. In the fourth and fifth lines, the zero frequency and those greater than mdiff are excluded. Finally, in the sixth line, Afgn and Aarf are estimated by adding 1 to the value returned by fminbnd().

### 2.7 The construction of whittle.m algorithm

We would like to warn the reader that in order to make this tutorial progressive, the steps are not ordered in the same way as in the whittle.m algorithm. Moreover, some algorithmic intricacies were unfolded in sub-steps, giving more lines of code than necessary to build the all-in-one whittle.m algorithm. To construct whittle.m, the reader should proceed as follows:

1. Begin with the header: function [Afgn, Aarf] = whittle(x), where x is the observation vector, Aarf is the exponent estimated using ARFIMA (0,*d*,0) theoretical power spectral density, and Afgn is the exponent estimated using fGn theoretical power spectral density.
2. Then paste the MATLAB codes in the following order:
  • **MATLAB code 1**: Periodogram estimation
  • **MATLAB code 8**: Optimization for fGn-based Whittle’s log-likelihood function
  • **MATLAB code 10**: If the observation vector is non-stationary, fGn-based Whittle’s likelihood
  • **MATLAB code 9**: Optimization for ARFIMA (0,*d*,0)-based Whittle’s log-likelihood function
  • **MATLAB code 11**: If the observation vector is non-stationary, ARFIMA-based Whittle’s likelihood
  • **MATLAB code 6**: Whittle’s log-likelihood MATLAB function with fGn theoretical PSD
  • **MATLAB code 7**: Whittle’s log-likelihood MATLAB function with ARFIMA (0,*d*,0) theoretical PSD

## 3 Whittle’s maximum likelihood performances

Now that all the steps have been described, we will test the performance of the whittle.m algorithm and, in particular, compare it to DFA, which is a widely used algorithm in fractal signal analysis. Regarding the first part of this tutorial, we propose to divide it into several sections. Section 3.1, *Test signals and generator biases*, describes the methodology applied to generate the signals used to test Whittle’s maximum likelihood estimator and describes the biases related to the two generators used. Section 3.2, *Which is the best estimator?*, evaluates and compares the three analysis methods using squared error values and thus determines which is of better quality. Then, the performance of ARFIMA-based Whittle’s likelihood depending on the signal length is discussed in Section 3.3, *Signal length*. Finally, Section 3.4, *Limitations and future studies*, outlines the misuse of fractal analysis on non-monofractal signals and the evolutions that can be made on this algorithm.

### 3.1 Test signals and generator biases

To test Whittle’s maximum likelihood estimator, we generated two sets of signals, one for each theoretical model (fGn/fBm and ARFIMA (0,*d*,0)). The first one, based on the fGn properties, is the Cholesky decomposition, whose algorithm is named cholfgn.m. The second one consists of ARFIMA (0,*d*,0) filtering, whose algorithm is named ARFIMA0d0.m. We thus generated, for each of these generators, 42 subsets of 120 signals of length *N* = 1,024 for a total of 5,040 signals for each generator. These 42 subsets correspond to 42 different α -values; the first value is 0.01 because the value 0 is excluded from the theoretical models, the following 19 values range from 0.05 to 0.95 by steps of 0.05, and the 21st value is 0.99 because the value 1 is also excluded from the models. The last 21 values are ranged in the same way from 1.01 to 1.99.

We then estimated $\hat{\alpha }$ using three analysis methods: fGn-based Whittle’s likelihood and ARFIMA-based Whittle’s likelihood, presented in this tutorial and implemented in the whittle.m function, and DFA, which is implemented in the DFA.m function. Note that the DFA algorithm we used is the improved version presented by Almurad and Delignières (2016). The algorithm of generation and analysis is presented in signal_generation_and_analysis.m, and the results are saved in generatedsignals.mat. The first striking result is the analysis time. It took 57.24 s to compute $\hat{\alpha }$ on the set of signals using the two Whittle’s functions, while it took 2052.30 s to compute $\hat{\alpha }$ on the set of signals using DFA. In sum, the analysis for Whittle’s function is approximately 70 times faster than DFA. We conducted the analysis on a Windows laptop equipped with an Intel i77700HQ processor, 16 GB of RAM, an Nvidia GTX 1050 graphics card, and a 512 GB SSD hard drive using MATLAB version 2019a.

**FIGURE 5:**
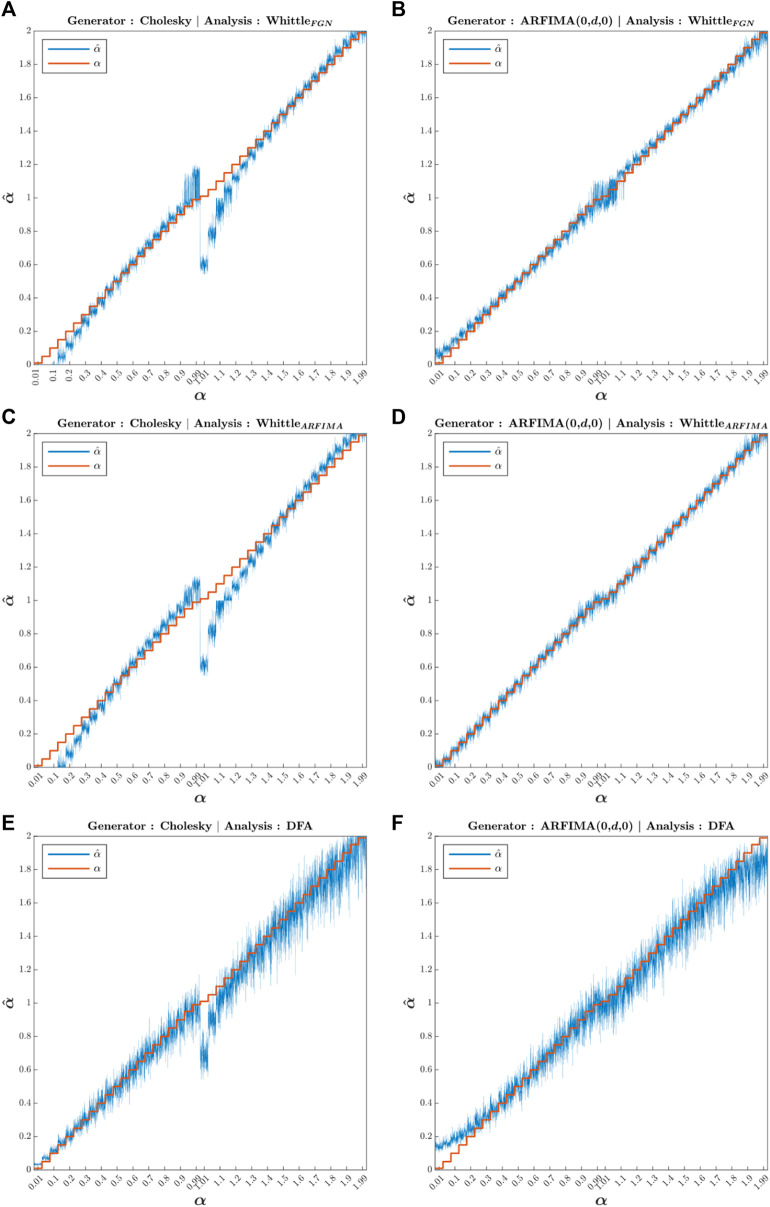
Whole set of $\hat{\alpha }$ estimated. On the *x*-axis, α is the true value of the exponents, the values computed in the generator. On the *y*-axis, the estimated values of $\hat{\alpha }$ computed by the corresponding analysis method are located. Red curves represent true alpha values α , and blue curves represent the estimated alpha values $\hat{\alpha }$ . The first column **(A,C,E)** corresponds to the signals generated via the Cholesky method, and the second column **(B,D,F)** corresponds to the signals generated via ARFIMA filtering. The first row **(A,B)** presents the $\hat{\alpha }$ values computed using fGn-based Whittle’s maximum likelihood estimator, the second row **(C,D)** presents those computed using ARFIMA-based Whittle’s maximum likelihood estimator, and the third row **(E,F)** presents the $\hat{\alpha }$ values computed using DFA.

Figure 5 shows the whole set of computed $\hat{\alpha }$ . This figure is arranged in two columns corresponding to the cholfgn and ARFIMA0d0 generators and in three rows corresponding to the three analysis methods: fGn-based Whittle’s maximum likelihood estimator, ARFIMA-based Whittle’s maximum likelihood estimator, and DFA. The figure shows that the cholfgn generator is strongly biased around the frontier between noise and motion (for α=1 ). We had already encountered this phenomenon in the study by Roume et al. (2019) using the Davies and Harte algorithm and can easily conclude that for generators based on the autocorrelation function of fGn, the integration of fractional Gaussian noises with an α close to 0 to obtain fractional Brownian motions with an α close to 1 is a technique that does not work properly. However, we can observe that this technique holds relatively well when the signals have been generated via ARFIMA filtering, as shown by the continuity between noise and motion observed in the bottom row. In addition, we will rely on the ARFIMA0d0 generator to estimate the efficiency of the analysis methods in the following section.

Type Fig5_Genbiases in the command window to access Figure 5.

### 3.2 Which is the best estimator?

In this section, we compare the efficiency of fGn-based Whittle’s likelihood, ARFIMA-based Whittle’s likelihood, and DFA. To assess the efficiency of these analysis methods, one must account for both their bias and variance. We therefore calculated Mean Squared Error (MSE), which is defined as the average of the squared error values. These analysis methods, being estimators in the sense of statistics, MSE can also be written as the sum of the variance and the squared bias of the values of $\hat{\alpha }$ , allowing us to directly compare their quality. Thus, when comparing two estimators, the estimator characterized by the smallest MSE value can be considered better.

**FIGURE 6:**
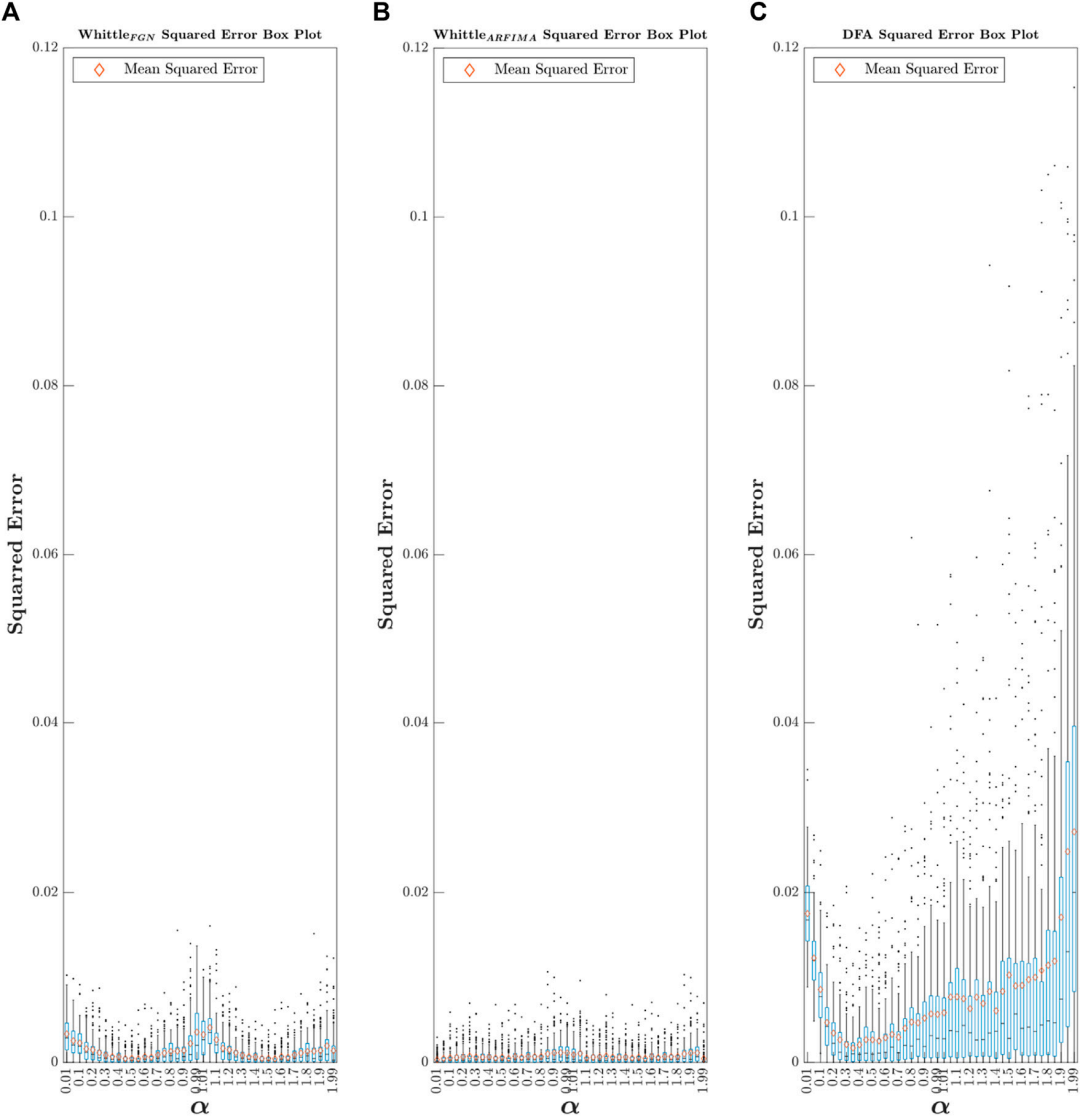
Box plot of $\hat{\alpha }$ squared error values obtained via fGn-based Whittle’s likelihood **(A)**, ARFIMA-based Whittle’s likelihood **(B)**, and DFA **(C)**. The lower and upper edges of the boxes represent the 25 and 75 percentiles, respectively. The horizontal black line represents the median. The whiskers extend to the most extreme points not considered as outliers. The outliers are plotted as individual points. The orange diamond represents the MSE value.

First, for each estimator, we calculated the 5,040 (42 α -values × 120 signals) squared error values, which were then averaged. We obtained an MSE of 0.0014±0.0019 for fGn-based Whittle’s likelihood^4^, 0.0007±0.0011 for ARFIMA-based Whittle’s likelihood, and 0.0078±0.0127 for DFA. We performed a one-way ANOVA that confirmed the significant differences between these groups (*F* (2,15,117) = 1,385.8; *p* < 0.001; *η* ^2^ = .15). Thus, ARFIMA-based Whittle’s likelihood is better than fGn-based Whittle’s likelihood, which itself is a better estimator than DFA. To go beyond the reductionist nature of this elementary comparison, a box plot of all the squared error values is constructed, as shown in Figure 6.

Type Fig6_SquarredError in the command window to access Figure 6.

In line with the results of the comparison of the MSE values, the first observation that can be made is that regardless of the value of α , the ARFIMA method is characterized by a lower median error than the other two analysis methods, as well as by a lower dispersion of these errors. We can also observe biases that could already be predicted from Figure 5; for example, for the fGn-based Whittle’s likelihood, the overestimation bias for α is less than 0.3, and the high variance around α is equal to 1. For DFA, the overestimation bias for α is less than 0.3, the underestimation bias for α is greater than 1.8, and there is a simultaneous increase between the variance of $\hat{\alpha }$ and the true α value.

### 3.3 Signal length

In this section, we discuss the performance of ARFIMA-based Whittle’s likelihood depending on signal lengths. Fractal analysis, like DFA, often requires a large series (N > 500) to provide reliable alpha estimates (Damouras et al., 2010; Phinyomark et al., 2020). This can lead to experimental issues when working with physiological signals, such as the difficulty of older adults to walk for more than 5 min, which is between 100 and 250 strides (Moon et al., 2016; Phinyomark et al., 2020).

**FIGURE 7:**
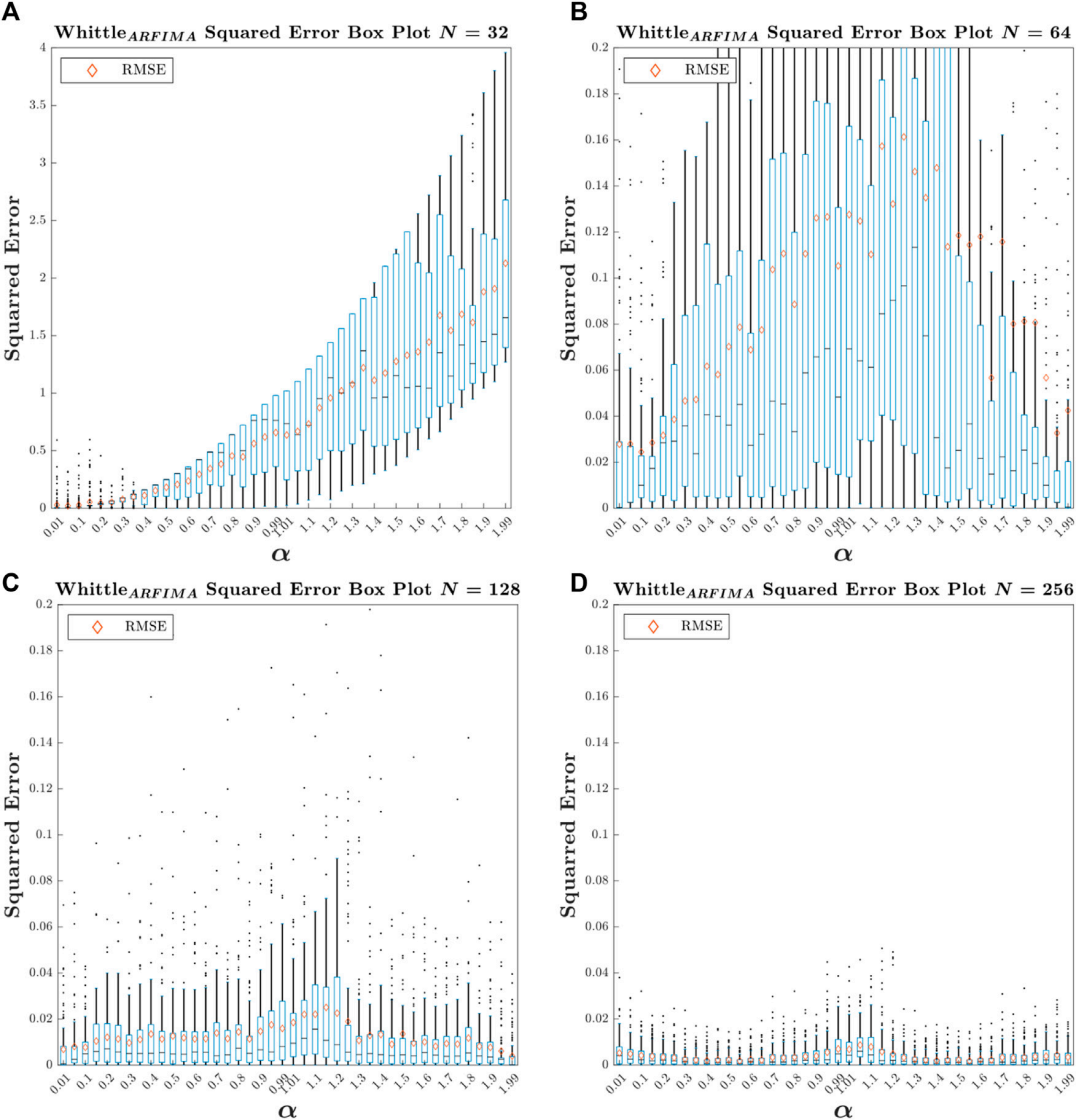
Box plot of $\hat{\alpha }$ squared error values obtained using ARFIMA-based Whittle’s likelihood for four sets of length: 32 **(A)**, 64 **(B)**, 128 **(C)**, and 256 **(D)**. The lower and upper edges of the boxes represent the 25 and 75 percentiles, respectively. The horizontal black line represents the median. The whiskers extend to the most extreme points not considered as outliers. The outliers are plotted as individual points. The orange diamond represents the MSE value. The vertical scale of the top left graph is 20 times larger than the other three panels.

To assess the performance, we first made four sets of signals by reducing the length of tsarf from 1 to *N*, with the following four *N* values: 32, 64, 128, and 256. We then estimated $\hat{\alpha }$ for these four sets and computed MSE. We obtained an MSE value of 0.7709±0.7954 for *N* = 32, 0.0884±0.1540 for *N* = 64, 0.0124±0.0207 for *N* = 128, and 0.0036±0.0053 for *N* = 256. Similar to Figure 6, the box plot of $\hat{\alpha }$ squared error values for the four reduced sets is constructed, as shown in Figure 7.

Type Fig7_SignalLength in the command window to access Figure 7.

The first observation that can be made from this figure is that the analysis method provides unreliable estimates for lengths *N* = 32 and 64. The second is that for *N* = 256, the ARFIMA-based Whittle’s likelihood gives more reliable estimates than DFA with *N* = 1,024. Finally, for *N* = 128, this method gives slightly less reliable estimates than DFA for 1,024 points.

In addition, it can be observed that, particularly for *N* = 128 and *N* = 256, the errors are maximized to approximately α = 1. These errors are linked to misclassification of signals as stationary or non-stationary. This is even more predominant for signals with an α exponent just above 1 for *N* = 128, although it should be noted that these signals are truncated and the variation in mean and standard deviation was distributed over the entire original signal. It would be interesting to add a size variable to the generated signals in future studies.

For further comparison, we calculated the value of MSE for $\hat{\alpha }$ obtained with DFA for *N* = 512 points, which gives 0.0203 ± 0.355, i.e., approximately twice the value of MSE for ARFIMA-based Whittle’s likelihood with *N* = 128 (0.0124 ± 0.0207). Considering that the variability of the version of DFA we use is optimized, for *N* = 512, the variability is smaller than that of the original DFA with *N* =1,024 (Almurad and Delignières, 2016), and given that several studies using the original DFA with *N* lengths of approximately 1,000 data points have given elegant results, it is reasonable to predict that an $\hat{\alpha }$ value estimated via ARFIMA-based Whittle’s likelihood on a series of 128 points could be acceptable even if not optimal.

### 3.4 Limitations and future studies

During the development of this tutorial, we tested Whittle’s maximum likelihood estimator on several physiological signals, including those made available by J. Hausdorff on the PhysioNet platform (Goldberger et al., 2000; Hausdorff, 2001). The results obtained are summarized in Table 2.

**TABLE 2:** Comparison of $\hat{\alpha }$ estimated via ARFIMA-based Whittle’s likelihood and DFA on gait data made available by J. Hausdorff on the PhysioNet platform (Goldberger et al., 2000; Hausdorff, 2001). The results highlighted in red correspond to anti-persistent series, i.e., with α lower than 0.5.

		Mean	SD	s1	s2	s3	s4	s5	s6	s7	s8	s9	s10
Slow	Whittle	0.96	0.06	0.99	0.82	0.94	0.99	1.00	0.95	1.04	1.01	0.98	0.91
	DFA	0.99	0.08	1.02	0.86	1.02	0.99	1.01	0.92	1.08	1.11	0.99	0.87
Normal	Whittle	0.89	0.05	0.91	0.80	0.96	0.85	0.84	0.82	0.93	0.91	0.93	0.91
	DFA	0.92	0.07	0.96	0.87	0.99	0.92	0.79	0.88	0.94	0.91	1.02	0.91
Fast	Whittle	0.93	0.05	0.94	0.95	0.99	0.85	1.00	0.95	0.91	0.94	0.85	0.95
	DFA	0.97	0.08	0.89	0.96	1.06	0.83	1.08	1.04	0.95	1.02	0.94	0.98
MetSlow	Whittle	0.46	0.26	0.52	0.74	0.21	0.39	0.02	0.49	0.68	0.13	0.67	0.71
	DFA	0.32	0.19	0.28	0.59	0.15	0.17	0.12	0.35	0.55	0.13	0.33	0.54
MetNorm	Whittle	0.60	0.20	0.68	0.47	0.74	0.55	0.11	0.54	0.73	0.69	0.76	0.72
	DFA	0.38	0.18	0.51	0.31	0.30	0.23	0.08	0.27	0.65	0.54	0.57	0.39
MetFast	Whittle	0.58	0.18	0.58	0.42	0.70	0.80	0.21	0.44	0.66	0.71	0.69	0.60
	DFA	0.37	0.16	0.31	0.29	0.38	0.64	0.11	0.22	0.59	0.33	0.38	0.47

Under the conditions without metronome (slow, normal, and fast), ARFIMA-based Whittle’s likelihood and DFA provide similar results for the mean $\hat{\alpha }$ , but a slightly higher standard deviation was observed for DFA. This result becomes very interesting under metronome conditions, especially when the ARFIMA-based Whittle’s likelihood and DFA provide diametrically opposed results, the first detecting persistence ( $\hat{\alpha }$ > 0.5) and the second detecting anti-persistence ( $\hat{\alpha }$ < 0.5).

**FIGURE 8:**
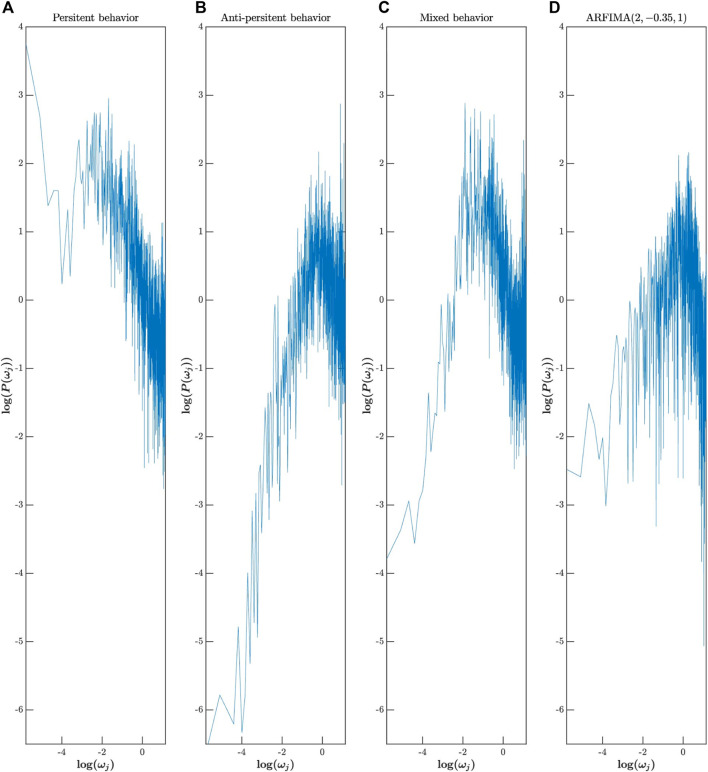
log-power spectral density of Hausdorff data rearranged into three groups: persistent behavior **(A)**, anti-persistent behavior **(B)**, and mixed behavior **(C)**. log-power spectral density of an artificial ARFIMA(p,d,q) signal with parameters (2, −0.35.1) generated using ARFIMApdq.m **(D)**.

To understand why we obtained these results, we grouped the different series into three groups: the first group, “persistent behavior,” assembles the series for which DFA and Whittle’s likelihood analysis provide an $\hat{\alpha }$ greater than 0.5. In the second group, “anti-persistent behavior,” we group the time series for which both DFA and Whittle likelihood provide an $\hat{\alpha }$ below 0.5. Finally, in the third group, “mixed behavior,” we group the series with $\hat{\alpha }$ lower than 0.5 from DFA and $\hat{\alpha }$ higher than 0.5 from Whittle’s likelihood. Then, we estimated the power spectra using the periodogram method, and finally, we averaged these spectra for each group. These averaged spectra are presented in the first three columns in Figure 8, and the data are saved in spectralbehavior. mat.

Type Fig8_SpectralBehavior in the command window to access Figure 8.

In the first two columns, when the Whittle’s and DFA methods provided similar $\hat{\alpha }$ (i.e., both greater than 0.5 for the first column and both lower than 0.5 for the second column), we observe that the power spectrum is dominated by a trend that is persistent in the first column and anti-persistent in the second column. In the third column, when Whittle’s and DFA provided opposite results, we can observe a mixed behavior where the low frequency part of the spectrum is dominated by anti-persistence, while the high frequencies are dominated by persistence.

This non-monotonicity of the spectrum in the logarithmic space, which was already observed by Torre and Delignières (2008), is not predicted in the two models, fGn/fBm and ARFIMA (0,*d*,0); therefore, we cannot apply these models, from a theoretical point of view, on a signal being characterized by such a spectrum. However, the ARFIMA (*p*,*d*,q) model allows the creation of non-monotonic spectra, as shown in the fourth column in Figure 8. It will therefore be interesting to improve the method that we have presented so far by incorporating in Equation 4 the AR and MA components known as short-memory components, all of which will give a theoretical spectrum with three parameters:

$$T_{ARFIMA}^{\prime }\left(\omega_{j};\varphi ,d,\theta \right)=\frac{1}{2\pi }{\left(2\sin\frac{\omega_{j}}{2}\right)}^{-2d}\frac{{\left|\theta \left(e^{-i\omega_{j}}\right)\right|}^{2}}{{\left|\varphi \left(e^{-i\omega_{j}}\right)\right|}^{2}},$$ (7)

where

• $\varphi \left(e^{-i\omega_{j}}\right)=1-\sum_{j=1}^{p}\varphi \left(j\right)e^{-i\omega_{j}j}$ is the autoregressive (AR) component.
• $\theta \left(e^{-i\omega_{j}}\right)=\sum_{j=0}^{q}\theta \left(j\right)e^{-i\omega_{j}j}$ is the moving average (MA) component.

Finally, it will be interesting to compare the proposed method in this tutorial with Higuchi’s fractal dimension method that Phinyomark et al. (2020) has shown to be better than DFA, which is on our agenda for future work.

## 4 Conclusion

The long-term dependency structures, or fractal structures, present in biological signals inform about the complexity of the system that produced these signals. With age or disease, this structure tends to be altered, making the fractal exponent *H* an interesting predictor in the domain of healthcare. However, the length of the signal required by current analysis methods to properly estimate the *H* exponent requires long series that are difficult to perform for people with motor impairments caused by advanced age or pathologies. In this tutorial, we described the steps to implement an analysis method based on Whittle’s approximation; then, we showed that this estimator was of better quality than the popular DFA, allowing for a reliable estimation of the *H* exponent for small series adapted to people who cannot perform physical activities over long periods.

## Data Availability

The datasets presented in this study can be found in online repositories. The names of the repository/repositories and accession number(s) can be found at: The MATLAB codes and datasets generated and analyzed for this study are deposited in a GitHub repository at https://github.com/clementroume/Whittle_maximum_likelihood_estimator_tutorial.

## Footnotes

1 György Inzelt (2022). ARFIMA(p,d,q) estimator (https://www.mathworks.com/matlabcentral/fileexchange/30238-arfima-p-d-q-estimator), MATLAB Central File Exchange. Retrieved July 12, 2022.

2 MathWorks Help Center (2021). periodogram. https://www.mathworks.com/help/signal/ref/periodogram.html

3 MathWorks Help Center (2021). fminbnd. https://www.mathworks.com/help/matlab/ref/fminbnd.html

4 The value displayed after the ± symbol is the standard deviation of the squared error of α.
